# Dopaminergic Modulation of Conscientiousness: *DRD2* rs1799732 and Personality Traits in Elite Mixed Martial Arts Athletes

**DOI:** 10.3390/genes16060720

**Published:** 2025-06-18

**Authors:** Milena Lachowicz, Remigiusz Recław, Krzysztof Chmielowiec, Jolanta Chmielowiec, Kinga Łosińska, Aleksandra Suchanecka, Jolanta Masiak, Anna Grzywacz

**Affiliations:** 1 Department and Clinic of Oncology and Radiotherapy, Medical University of Gdansk, ul. M. Skłodowskiej-Curie 3a, 80-210 Gdansk, Poland; milena.lachowicz@awf.gda.pl; 2 Department of Psychology, Gdansk University of Physical Education and Sport, Kazimierza Górskiego 1 St., 80-336 Gdansk, Poland; 3 Independent Laboratory of Behavioral Genetics and Epigenetics, Pomeranian Medical University in Szczecin, Powstancow Wielkopolskich 72 St., 70-111 Szczecin, Poland; remigiusz.reclaw@pum.edu.pl (R.R.); aleksandra.suchanecka@pum.edu.pl (A.S.); 4 Department of Medical Sciences and Public Health, Gdansk University of Physical Education and Sport, Kazimierza Gorskiego 1 St., 80-336 Gdansk, Poland; kinga.losinska@awf.gda.pl; 5 Department of Hygiene and Epidemiology, Collegium Medicum, University of Zielona Góra, 28 Zyty St., 65-046 Zielona Góra, Poland; chmiele@vp.pl (K.C.); chmiele1@o2.pl (J.C.); 6 Second Department of Psychiatry and Psychiatric Rehabilitation, Medical University of Lublin, 1 Głuska St., 20-059 Lublin, Poland; jolantamasiak@wp.pl

**Keywords:** personality traits, DRD2, athletes, conscientiousness, gene–environment interaction, MMA athletes, dopamine D2 receptor, psychogenetics

## Abstract

Background: Personality traits, particularly Conscientiousness, are recognised as crucial psychological factors contributing to success in elite-level athletes. Emerging evidence suggests that individual differences in these traits are influenced by environmental exposure and genetic variation, especially within the dopaminergic system. The *DRD2* promoter polymorphism rs1799732, which affects dopamine D2 receptor expression, may modulate goal-directed behaviour and self-regulation traits. Methods: This study included 323 participants (141 elite mixed martial arts (MMA) athletes and 182 non-athlete controls). Participants completed the NEO Five-Factor Inventory (NEO-FFI). Genotyping for the *DRD2* rs1799732 polymorphism was conducted using real-time PCR. Group comparisons and two-way ANOVA were used to assess genotype–phenotype associations and gene × environment interactions. Results: Athletes scored significantly higher on Conscientiousness than controls. A significant main effect of the DRD2 rs1799732 genotype and a genotype × group interaction were observed for Conscientiousness. Specifically, athletes with the ins/ins genotype exhibited the highest levels of Conscientiousness, whereas individuals with the del/del genotype showed the lowest scores. No significant associations were found for other personality traits. Conclusions: These findings suggest that the DRD2 promoter polymorphism rs1799732 moderates the expression of Conscientiousness, particularly under the structured and demanding conditions experienced by elite athletes. Our results support a gene × environment interaction model, highlighting the importance of considering genetic predispositions in high-performance environments. These insights may inform personalised psychological support strategies tailored to athletes’ genetic profiles, enhancing motivation, self-regulation and long-term athletic development.

## 1. Introduction

Personality traits have long been recognised as critical psychological factors that influence daily functioning and performance in highly demanding environments such as competitive sports. In particular, Conscientiousness, a significant dimension of the Five-Factor Model (FFM) [1], has been strongly associated with success in elite athletic contexts [2,3]. This trait encompasses self-discipline, orderliness, persistence, and the ability to pursue long-term goals—qualities essential for sustaining the rigorous demands of professional athletes.

However, while the psychological significance of Conscientiousness is well established, its biological basis remains less thoroughly investigated. It is increasingly acknowledged that individual differences in personality traits are not solely shaped by life experiences but also have a substantial genetic basis [4]. Indeed, integrating genetic, neurobiological, and psychological perspectives is crucial for a more complete and comprehensive understanding of the mechanisms underpinning behaviour.

Among the biological systems implicated in personality development, the dopaminergic system stands out for its central role in motivation, reward sensitivity, goal-directed behaviour, and executive functioning [5,6]. Dopamine transmission influences individuals’ responses to incentives, plans actions, and maintains effort toward distant goals. Notably, alterations in dopamine signalling have been linked not only to psychiatric disorders but also to normal variation in traits such as Conscientiousness and impulsivity [7].

It is worth noting that among the numerous genes related to dopamine function, the dopamine D2 receptor gene (*DRD2*) has received particular attention. *DRD2* encodes the D2 subtype of dopamine receptors, predominantly expressed in the striatum and prefrontal cortex—brain regions critically involved in cognitive control and motivational regulation [8]. Within this gene, the promoter polymorphism rs1799732, characterised by an insertion/deletion (ins/del) variant, has been identified as functionally relevant. Studies suggest that the deletion allele may lead to reduced transcriptional activity, resulting in lower D2 receptor density [9,10].

The implications of such biological variation are undeniable: diminished executive control, reduced motivation to maintain goal-directed behaviours, and greater susceptibility to external influences have all been linked to the lower availability of D2 receptors [11]. Therefore, it is plausible to hypothesise that the *DRD2* rs1799732 polymorphism may contribute to individual differences in traits such as Conscientiousness, especially under conditions of high environmental demand.

Although previous research has examined associations between dopaminergic gene polymorphisms (e.g., *DRD2*, *COMT*, *DAT1*) and personality traits [12,13], studies specifically addressing the *DRD2* rs1799732 polymorphism in the context of athletic populations are still lacking. Given the intensive reinforcement structures, goal-oriented practices, and cognitive–emotional demands inherent in elite athletes, such settings may amplify the expression of genetically mediated traits, serving as a real-world model for studying gene–environment interactions.

Emerging evidence supports the gene–environment interaction (G × E) framework, suggesting that genetic influences on behaviour are not static but may vary depending on environmental exposures [14]. In the case of high-level athletes, the structured and challenging environment could act as a catalyst that enhances or attenuates the influence of genetic predispositions on personality traits [15].

Thus, the primary aim of the present study was to examine the association between the *DRD2* promoter polymorphism rs1799732 and personality traits, with a particular focus on Conscientiousness, in a group of elite mixed martial arts (MMA) athletes compared to non-athlete controls. Specifically, we hypothesised that the following:

Athletes would display higher levels of Conscientiousness than controls.

The *DRD2* rs1799732 genotype would be associated with differences in Conscientiousness.

A significant interaction between genotype and group (athletes vs. controls) would emerge, reflecting the environmental moderation of genetic effects.

Through this approach, we aim to contribute to the growing field of psychogenetics of personality in sports, shedding light on how biological factors interact with environmental contexts to shape individual profiles conducive to elite performance.

## 2. Materials and Methods

### 2.1. Participants

This study included 323 volunteers, comprising 141 professional athletes (males = 92, 65%; females = 49, 35%; mean age = 22.7 years, SD = 6.02) and 182 non-athlete controls (males = 146, 80%; females = 36, 20%; mean age = 21.9 years, SD = 3.88). The Bioethics Committee for Clinical Research of the Regional Medical Society approved the study protocol in Szczecin, Poland (Protocol No. 13/KB/VI/2016, issued on 8 December 2016). All participants provided written informed consent before enrolment.

Data collection was conducted at the Independent Laboratory of Behavioral Genetics and Epigenetics. Both the athlete and control groups underwent psychiatric evaluation using the Mini International Neuropsychiatric Interview (MINI) [16] and the NEO Five-Factor Inventory (NEO-FFI) [17].

The athlete group consisted of participants actively competing in national or international tournaments across various disciplines, including karate (n = 6), judo (n = 17), boxing (n = 19), mixed martial arts (MMA) (n = 31), jiu-jitsu (n = 5), kickboxing (n = 8), volleyball (n = 5), handball (n = 5), ice hockey (n = 24), triathlon (n = 1), basketball (n = 4), and football (n = 16). All athletes had participated in competitive events within the past year and maintained systematic training for at least five years. Control participants were not participating in sports professionally or semi-professionally. All participants were recruited in Poland and were of Caucasian origin.

### 2.2. Measures

The Mini International Neuropsychiatric Interview (MINI) is a structured diagnostic tool designed to assess psychiatric disorders according to the criteria of the DSM-IV and ICD-10. The MINI was used to verify participants’ neuropsychiatric health.

Personality traits were assessed using the NEO Five-Factor Inventory (NEO-FFI), which evaluates five major domains: Neuroticism (anxiety, hostility, depression, self-consciousness, impulsivity, vulnerability to stress), Extraversion (warmth, sociability, assertiveness, activity level, excitement seeking, positive emotions), Openness to Experience (fantasy, aesthetics, feelings, actions, ideas, values), Agreeableness (trust, straightforwardness, altruism, compliance, modesty, tenderness), and Conscientiousness (competence, order, dutifulness, achievement striving, self-discipline, deliberation).

Scores were converted to standardised sten scores based on Polish adult norms according to the participants’ age and sex. According to these norms, sten scores 1–2 indicate very low results, 3–4 low, 5–6 average, 7–8 high, and 9–10 very high results.

### 2.3. Genotyping

Genomic DNA was extracted from venous blood samples using a commercially available protocol (QIAamp Blood DNA Mini Kit, QIAGEN, Hilden, Germany).

The genotyping of the *DRD2* promoter polymorphism rs1799732 was conducted via real-time polymerase chain reaction on the LightCycler 480II instrument (Roche Diagnostics, Basel, Switzerland) with the LightSNiP (TiBMolBiol, Berlin, Germany) oligonucleotide assay under the following conditions: initial denaturation (95 °C/10 min), followed by 45 cycles of denaturation (95 °C/10 s), annealing (60 °C/10 s), and elongation (72 °C/15 s). The melting curve of the PCR products was generated with denaturation (95 °C/30 s) and cooling (40 °C/2 min), followed by heating to 75 °C at a 1.5 °C/s. rate. The reaction was terminated by 30 s of cooling at 40 °C. Fluorescence signals were plotted against temperature to generate melting curves for each sample, allowing the identification of the genotypes based on characteristic peak temperatures. *DRD2* rs1799732 peaks were read at 56.54 °C for the C insertion allele and 62.85 °C for the C deletion allele. Negative (control) samples were subjected to examination across each reaction plate. Additionally, 10% of the samples were duplicated with 100% accuracy.

### 2.4. Statistical Analysis

The distribution of genotypes was tested for compliance with Hardy–Weinberg equilibrium (HWE) using the online HWE calculator (https://wpcalc.com/en/equilibrium-hardy-weinberg/, accessed on 12 December 2024).

Relationships between the *DRD2* rs1799732 polymorphism, athlete status, and personality traits were examined using multivariate analysis of variance (ANOVA) with the following model: NEO-FFI trait × genotype (del/del vs. ins/ins + del/del) × group (athletes vs. controls) and their interactions.

The assumption of homogeneity of variance was verified using Levene’s test (*p* > 0.05). Since the distributions of the analysed variables were non-normal, comparisons of NEO-FFI traits between groups were additionally conducted using the nonparametric Mann–Whitney U-test.

Genotype frequency differences between athletes and controls were assessed using the chi-square test. All statistical analyses were performed using STATISTICA 13 software (TIBCO Software Inc., Palo Alto, CA, USA) for Windows (Microsoft Corporation, Redmond, WA, USA).

## 3. Results

The *DRD2* promoter polymorphism rs1799732 genotype distributions were consistent with Hardy–Weinberg equilibrium (HWE) in both the athlete and control groups. Detailed data are presented in Table 1.

Genotype distributions of the *DRD2* promoter polymorphism rs1799732 were tested for Hardy–Weinberg equilibrium (HWE) using a chi-square test. No significant deviations from HWE were observed in either athletes (*p* = 0.3264) or controls (*p* = 0.4203).

No statistically significant differences were observed in *DRD2* rs1799732 genotypes or allele frequency distribution between the athlete and control groups. Detailed frequencies are presented in Table 2.

Chi-square tests revealed no significant differences in the distribution of *DRD2* rs1799732 genotypes (χ^2^ = 2.541; *p* = 0.2806) or allele frequencies (χ^2^ = 0.847; *p* = 0.3572) between athletes and controls.

Table 3 presents the means and standard deviations for NEO-FFI personality trait scores in athletes and control participants.

Group differences in NEO-FFI personality traits were analysed using the Mann–Whitney U-test. Athletes scored significantly higher than controls on Conscientiousness (Z = 4.031; *p* = 0.0001). No significant group differences were observed for Neuroticism, Extraversion, Openness to Experience, and Agreeableness.

The results of the two-way ANOVA (group × *DRD2* rs1799732 genotype) for the NEO-FFI personality traits are summarised in Table 4. Due to the small number of recessive del/del homozygotes, this group was combined with ins/del heterozygotes to create the ins/del + del/del group. A particular focus was placed on the Conscientiousness trait, where significant effects were observed.

**Figure 1:**
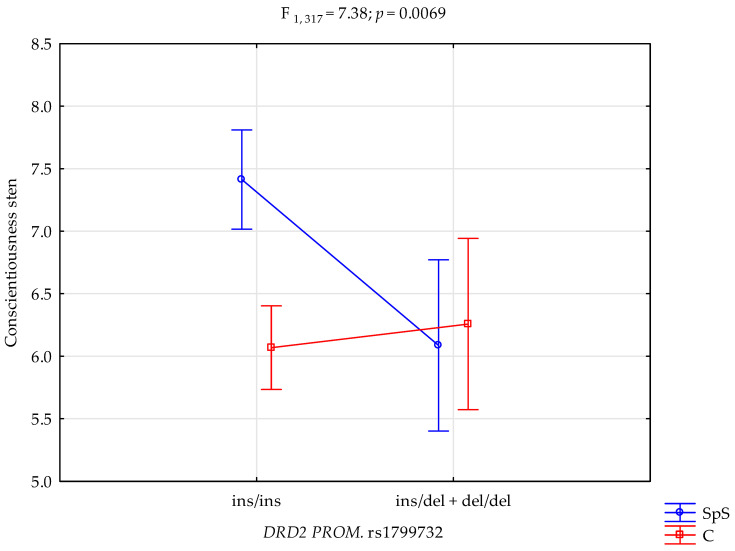
Interaction between group status (athletes; SPS vs. controls; C) and *DRD2* rs1799732 genotypes on Conscientiousness sten scores.

A significant interaction was observed between the DRD2 rs1799732 genotype and group status (athletes vs. controls) on Conscientiousness scores (F_1,319_ = 7.38; *p* = 0.0069; Figure 1). Athletes carrying the ins/ins genotype demonstrated the highest Conscientiousness scores, while those with the ins/del + del/del genotype exhibited the lowest scores. The interaction effect suggests that the relationship between genotype and Conscientiousness is moderated by group membership (athletic involvement).

The results of the post hoc analysis (LSD test) exploring group and genotype differences in Conscientiousness are presented in Table 5.

## 4. Discussion

The present study investigated the association between the *DRD2* promoter polymorphism rs1799732 and personality traits, particularly Conscientiousness, in a sample of elite mixed martial arts (MMA) athletes and non-athlete controls. Although genotype and allele frequencies did not significantly differ between groups, significant genotype–phenotype associations were observed for Conscientiousness, supporting this study’s main hypotheses.

Consistent with previous research [2,3], athletes scored significantly higher on Conscientiousness compared to controls. This finding highlights the critical role of self-discipline, persistence, and goal-oriented behaviour in the demanding context of elite athletes, particularly in combat disciplines where cognitive control and emotional regulation are pivotal for success. These results align with the existing literature showing that high-performing athletes often exhibit enhanced executive functioning and self-regulatory capacities, essential for maintaining focus, managing stress, and adhering to rigorous training regimens. Importantly, MMA athletes represent a unique population due to their sport’s intense physical, mental, and emotional demands. Success in MMA requires physical prowess, strategic thinking, emotional resilience, and exceptional time management, all of which fall under the umbrella of Conscientiousness. Our findings suggest that the interplay between environmental demands and dopaminergic function may further shape these traits at both behavioural and neurobiological levels.

Importantly, the current study extends previous findings by demonstrating that genetic variation in the *DRD2* promoter region significantly influences the expression of Conscientiousness, particularly in an environment characterised by structured training and high-performance demands. Specifically, individuals with the ins/ins genotype scored considerably higher on Conscientiousness than those with del/del or ins/del genotypes, particularly within the athlete group. The observed genotype × environment interaction supports the notion that genetic predispositions are not uniformly expressed across all contexts but may be potentiated by environmental conditions, in line with the differential susceptibility hypothesis [14].

These findings are consistent with prior work suggesting that dopamine D2 receptor availability influences executive function, self-control, and reward-based learning [5,7]. Studies using positron emission tomography (PET) have demonstrated that a higher D2 receptor density in the prefrontal cortex is associated with improved performance in tasks requiring sustained effort and the inhibition of impulsive responses [18]. Thus, the higher levels of Conscientiousness observed in ins/ins carriers could reflect the enhanced dopaminergic modulation of cognitive control systems.

Interestingly, no significant genotype effects were observed for Neuroticism, Extraversion, Openness to Experience, or Agreeableness. This specificity echoes previous findings that dopaminergic genetic variation predominantly impacts the executive and motivational dimensions of personality, rather than broad affective or interpersonal traits [19]. In this light, the current study reinforces the emerging view that dopaminergic genes are selectively involved in personality facets linked to self-regulation and goal pursuit.

Our results partially align with earlier genetic studies linking the *DRD2 TaqIA* polymorphism to Conscientiousness and other executive-related traits [12,20]. However, different *DRD2* variants may exert differential effects depending on their location (promoter vs. coding region) and functional consequences. Nevertheless, our findings and those of prior studies point toward a converging model where dopaminergic system efficiency underlies stable individual differences in self-regulatory capacities.

It is worth highlighting that the interaction between genetic predisposition and environmental exposure emerged most clearly in athletes. This observation supports models proposing that extreme or structured environments, such as those encountered in elite athletes, amplify genetic differences [21]. Such differences may remain latent in less demanding environments or exert minimal behavioural influence.

From a practical standpoint, these findings offer potential implications for the psychological management of MMA athletes. Coaches and sport psychologists may benefit from considering individual differences in dopaminergic function when designing training programs, setting goals, and providing psychological support. Athletes with the del/del or ins/del genotype may require more structured guidance, external reinforcement, and tailored motivation strategies to achieve optimal self-regulation and long-term performance outcomes. In contrast, ins/ins carriers may thrive under autonomous, self-directed approaches that emphasise personal responsibility and intrinsic motivation.

Several limitations of the present study warrant consideration. First, the cross-sectional design precludes causal inferences regarding the relationship between the *DRD2* genotype and Conscientiousness expression. Second, although the overall sample size was satisfactory, the number of del/del carriers was small, limiting the statistical power for some genotype comparisons. Third, this study focused exclusively on MMA athletes, who may represent a particularly self-selected group with extreme psychological profiles. Lastly, we did not collect information regarding the intensity of training, years of experience, or competitive level among athletes, and we did not control for these factors in our analysis.

Future research should replicate these findings in other athletic disciplines, include mixed-sex samples, and apply longitudinal designs to investigate how genetic predispositions interact with training experience over time. Incorporating neuroimaging methods (e.g., D2 receptor binding potential) and polygenic approaches would also deepen our understanding of the complex interplay between genes, brain function, personality, and performance outcomes.

In conclusion, the present study provides novel evidence that the *DRD2* promoter polymorphism rs1799732 moderates Conscientiousness, particularly within elite athletes’ structured, high-demand environments. These findings contribute to the growing literature on the biological bases of personality and underscore the importance of considering gene × environment interactions when examining psychological traits related to human achievement and adaptation.

## 5. Conclusions

The present study provides novel evidence that genetic variation in the dopamine D2 receptor gene, specifically the *DRD2* promoter polymorphism rs1799732, is associated with differences in Conscientiousness, particularly within elite athletic performance. Athletes carrying the ins/ins genotype exhibited significantly higher levels of Conscientiousness compared to those carrying del alleles, and a significant interaction between genotype and athlete status was observed.

These findings emphasise the importance of considering gene × environment interactions in studying personality traits, especially those related to self-regulation, persistence, and goal-directed behaviour. The results also highlight the potential contribution of dopaminergic function to the psychological characteristics essential for success in high-performance environments such as mixed martial arts.

Future research should expand upon these findings by incorporating larger and more diverse samples, applying longitudinal designs, and integrating neuroimaging and polygenic analyses to understand better the complex biological and environmental pathways influencing personality traits and athletic performance.

## Figures and Tables

**Table 1:** Hardy–Weinberg equilibrium for *DRD2* rs1799732 genotypes in athletes and control participants.

Hardy–Weinberg Equilibrium, Including Analysis for Ascertainment Bias		Observed (Expected)	Allele Freq	χ^2^ (*p*-Value)
*DRD2* rs1799732				
Athletes n = 141	ins/ins	106 (107.3)	p (ins) = 0.87 q (del) = 0.13	0.963 (0.3264)
	del/del	1 (2.3)		
	ins/del	34 (31.4)		
Controls n = 182	ins/ins	147 (146)	p (ins) = 0.90 q (del) = 0.10	0.649 (0.4203)
	del/del	3 (2)		
	ins/del	32 (34)		

*p*—statistical significance χ^2^ test.

**Table 2:** Distribution of *DRD2* rs1799732 genotypes and allele frequencies among athletes and control participants.

*DRD2* rs1799732					
	Genotypes			Alleles	
	ins/ins n (%)	del/del n (%)	ins/del n (%)	ins n (%)	del n (%)
Athletes n = 141	106 (75.18%)	1 (0.71%)	34 (24.11%)	246 (87.23%)	36 (12.77%)
Controls N = 182	147 (80.77%)	3 (1.65%)	32 (17.58%)	326 (89.56%)	38 (10.44%)
χ^2^ (*p*-value)	2.541 0.2806			0.847 (0.3572)	

n—number of subjects. *p*—tatistical significance.

**Table 3:** NEO Five-Factor Inventory (NEO-FFI) sten scores in athletes and control participants.

NEO Five-Factor Inventory	Athletes (n = 141)	Control (n = 182)	Z	(*p*-Value)
Neuroticism scale	4.86 ± 2.15	4.48 ± 1.96	1.296	0.1951
Extraversion scale	6.49 ± 1.85	6.63 ± 1.77	−0.8259	0.4088
Openness scale	4.84 ± 2.35	4.54 ± 1.62	1.0890	0.2761
Agreeability scale	5.96 ± 3.94	5.77 ± 1.94	−0.1147	0.9087
Conscientiousness scale	7.08 ± 2.07	6.10 ± 2.10	4.0314	0.0001 *

*p*, statistical significance with Mann–Whitney U-test; *n,* number of subjects; M ± SD, mean ± standard deviation; * statistically significant differences.

**Table 4:** Two-way ANOVA results for *DRD2* rs1799732 genotypes and NEO-FFI traits in athletes and controls.

NEO Five-Factor Inventory	Group	DRD2 rs1799732		ANOVA			
		ins/ins n = 253 (106 A + 147 C) M ± SD	ins/del + del/del n = 70 (34 + 1 A + 32 + 3 C) M ± SD	Factor	F (*p* Value)	ɳ^2^	Power (Alfa = 0.05)
Neuroticism scale	Athletes (Athl); n = 141	4.71 ± 2.11	5.31 ± 2.25	intercept Athl/control *DRD2* Athl/control × *DRD2*	F_1,319_ = 1155.76 (*p* < 0.0001) * F_1,319_ = 4.98 (*p* = 0.0264) * F_1,319_ = 0.36 (*p* = 0.5474) F_1,319_ = 2.53 (*p* = 0.1126)	0.784 0.015 0.001 0.008	1.000 0.604 0.092 0.354
	Control; n = 182	4.53 ± 1.99	4.26 ± 1.82				
Extraversion scale	Athletes (Athl); n = 141	6.52 ± 1.87	6.40 ± 1.80	intercept Athl/control *DRD2* Athl/control × *DRD2*	F_1,319_ = 2883.83 (*p* < 0.0001) * F_1,319_ = 1.10 (*p =* 0.2934) F_1,319_ = 0.11 (*p* = 0.7445) F_1,319_ = 0.66 (*p* = 0.4182)	0.900 0.003 0.0003 0.002	1.000 0.183 0.062 0.128
	Control; n = 182	6.58 ± 1.73	6.86 ± 1.96				
Openness scale	Athletes (Athl); n = 141	4.77 ± 2.52	5.03 ± 1.77	intercept Athl/control *DRD2* Athl/control × *DRD2*	F_1,319_ = 1263.17 (*p* < 0.0001) * F_1,319_ = 1.13 (*p =* 0.2884) F_1,319_ = 0.90 (*p* = 0.3435) F_1,319_ = 0.01 (*p* = 0.9999)	0.798 0.004 0.003 0.0001	1.000 0.185 0.157 0.050
	Control; n = 182	4.49 ± 1.61	4.74 ± 1.67				
Agreeability scale	Athletes (Athl); n = 141	6.16 ± 4.34	5.34 ± 2.30	intercept Athl/control *DRD2* Athl/control × *DRD2*	F_1,319_ = 819.58 (*p* < 0.0001) * F_1,319_ = 0.02 (*p =* 0.8781) F_1,319_ = 0.69 (*p* = 0.4053) F_1,319_ = 1.42 (*p* = 0.2348)	0.720 0.0001 0.002 0.004	1.000 0.053 0.132 0.221
	Control; n = 182	5.74 ± 2.01	5.89 ± 1.68				
Conscientiousness scale	Athletes (Athl); n = 141	7.41 ± 1.80	6.09 ± 2.48	intercept Athl/control *DRD2* Athl/control × *DRD2*	F_1,319_ = 2138.36 (*p* < 0.0001) * F_1,319_ = 4.42 (*p* = 0.0363) * F_1,319_ = 4.16 (*p* = 0.0423) * F_1,319_ = 7.38 (*p* = 0.0069) *	0.871 0.014 0.013 0.023	1.000 0.554 0.529 0.773
	Control; n = 182	6.07 ± 2.08	6.26 ± 2.24				

*—significant result; Athl, A—athletes; C—control; M ± SD—mean ± standard deviation.

**Table 5:** Post hoc test (Least Significant Difference) analysis of interactions between the athletes/control and *DRD2* rs1799732 and Conscientiousness sten score.

	{1} M = 7.41	{2} M = 6.09	{3} M = 6.07	{4} M = 6.26
Athletes ins/ins {1}		0.00108 *	0.00001 *	0.00433 *
Athletes ins/del + del/del {2}			0.96360	0.72786
Control ins/ins {4}				0.62565
Control ins/del + del/del {5}				

*—significant statistical differences; M—mean.

## Data Availability

The data presented in this study are available on request from the corresponding author. The data are not publicly available due to privacy concerns.

## References

[1] McCrae R.R., Costa P.T. (1992). Discriminant validity of NEO-PIR facet scales. J. Personal..

[2] Allen M.S., Greenlees I., Jones M. (2013). Personality in sport: A comprehensive review. Int. Rev. Sport Exerc. Psychol..

[3] McCormick A., Trew K. (2017). Personality and performance: Understanding the links. Personal. Individ. Differ..

[4] DeYoung C.G. (2010). Personality neuroscience and the biology of traits. Soc. Personal. Psychol. Compass.

[5] Wise R.A. (2004). Dopamine, learning and motivation. Nat. Rev. Neurosci..

[6] Berridge K.C., Robinson T.E. (1998). What is the role of dopamine in reward: Hedonic impact, reward learning, or incentive salience?. Brain Res. Rev..

[7] Buckholtz J.W., Meyer-Lindenberg A. (2012). Psychopathology and the human connectome: Toward a transdiagnostic model of risk for mental illness. Neuron.

[8] Savitz J., Ramesar R. (2004). Genetic variants implicated in personality: A review of the evidence and a model for future research. Mol. Psychiatry.

[9] Arinami T., Gao M., Hamaguchi H., Toru M. (1997). A functional polymorphism in the promoter region of the human dopamine D2 receptor gene is associated with schizophrenia. Hum. Mol. Genet..

[10] Persico A.M., Bird E.D. (1993). Dopamine receptor genes and psychiatric disorders: Is the D2 receptor gene implicated?. Mol. Psychiatry.

[11] Jocham G., Klein T.A., Neumann J., von Cramon D.Y., Reuter M., Ullsperger M. (2009). Dopamine D2 receptors and cognitive control: The influence of receptor availability on brain activation. Neuron.

[12] Niewczas M., Grzywacz A., Leźnicka Duniec K., Chmielowiec K., Chmielowiec J., Maciejewska Skrendo A., Ruzbarsky P., Masiak J., Czarny W., Cięszczyk P. (2021). Association between Polymorphism rs1799732 of DRD2 Dopamine Receptor Gene and Personality Traits among MMA Athletes. Genes.

[13] Felten A., Montag C., Kranczioch C., Markett S., Walter N.T., Reuter M. (2013). The DRD2 C957T polymorphism and the attentional blink—A genetic association study. Eur. Neuropsychopharmacol..

[14] Belsky J., Pluess M. (2009). Beyond diathesis stress: Differential susceptibility to environmental influences. Psychol. Bull..

[15] Choi S., Lee S., Huh I., Hwang H., Park T. (2020). HisCoM-G×E: Hierarchical Structural Component Analysis of Gene-Based Gene–Environment Interactions. Int. J. Mol. Sci..

[16] Cloninger C.R., Przybeck T.R., Svrakic D.M., Wetzel R.D. (1994). The Temperament and Character Inventory (TCI): A Guide to Its Development and Use.

[17] Costa P.T., McCrae R.R., Boyle G.J., Matthews G., Saklofske D.H. (2008). The Revised NEO Personality Inventory (NEO-PI-R). The SAGE Handbook of Personality Theory and Assessment: Volume 2—Personality Measurement and Testing.

[18] Jocham G., Klein T.A., Ullsperger M. (2011). Dopamine-mediated reinforcement learning signals in the striatum and ventromedial prefrontal cortex underlie value-based choices. J. Neurosci..

[19] Cools R., D’Esposito M. (2011). Inverted-U-shaped dopamine actions on human working memory and cognitive control. Biol. Psychiatry.

[20] Montag C., Jurkiewicz M., Reuter M. (2012). The role of the DRD2 C957T polymorphism in personality traits: Evidence from a German population sample. Behav. Genet..

[21] Manuck S.B., McCaffery J.M., Rao D.C. (2011). Gene–environment interaction. Annu. Rev. Psychol..

